# Population-Wide Depression Incidence Forecasting Comparing Autoregressive Integrated Moving Average and Vector Autoregressive Integrated Moving Average to Temporal Fusion Transformers: Longitudinal Observational Study

**DOI:** 10.2196/67156

**Published:** 2025-05-12

**Authors:** Deliang Yang, Yiyi Tang, Vivien Kin Yi Chan, Qiwen Fang, Sandra Sau Man Chan, Hao Luo, Ian Chi Kei Wong, Huang-Tz Ou, Esther Wai Yin Chan, David Makram Bishai, Yingyao Chen, Martin Knapp, Mark Jit, Dawn Craig, Xue Li

**Affiliations:** 1 Department of Medicine School of Clinical Medicine, Li Ka Shing Faculty of Medicine The University of Hong Kong Hong Kong China (Hong Kong); 2 Faculty of Science University of Hong Kong Hong Kong China (Hong Kong); 3 Department of Pharmacology and Pharmacy Li Ka Shing Faculty of Medicine The University of Hong Kong Hong Kong China (Hong Kong); 4 Department of Psychiatry Faculty of Medicine The Chinese University of Hong Kong Hong Kong China (Hong Kong); 5 School of Public Health Sciences University of Waterloo Waterloo, ON Canada; 6 School of Pharmacy Aston University Birmingham United Kingdom; 7 Advanced Data Analytics for Medical Science (ADAMS) Limited Hong Kong China (Hong Kong); 8 Institute of Clinical Pharmacy and Pharmaceutical Sciences College of Medicine National Cheng Kung University Tainan Taiwan; 9 Department of Pharmacy College of Medicine National Cheng Kung University Tainan Taiwan; 10 Laboratory of Data Discovery for Health (D24H) Hong Kong China (Hong Kong); 11 Division of Health Economics, Policy and Management School of Public Health, Li Ka Shing Faculty of Medicine The University of Hong Kong Hong Kong China (Hong Kong); 12 National Health Commission Key Laboratory of Health Technology Assessment Fudan University Shanghai China; 13 Care Policy and Evaluation Centre London School of Economics and Political Science London United Kingdom; 14 Department of Infectious Disease Epidemiology Faculty of Epidemiology and Population Health London School of Hygiene & Tropical Medicine London United Kingdom; 15 Department of Global and Environmental Health School of Global Public Health New York University New York, NY United States; 16 School of Public Health Li Ka Shing Faculty of Medicine University of Hong Kong Hong Kong China (Hong Kong); 17 Population Health Sciences Institute Faculty of Medical Sciences Newcastle University Newcastle United Kingdom

**Keywords:** population-wide depression incidence, depression incidence forecasting, structural break scenarios, electronic health records, machine learning, medical informatics, ARIMA, vector-ARIMA, temporal fusion transformers, deep learning

## Abstract

**Background:**

Accurate prediction of population-wide depression incidence is vital for effective public mental health management. However, this incidence is often influenced by socioeconomic factors, such as abrupt events or changes, including pandemics, economic crises, and social unrest, creating complex structural break scenarios in the time-series data. These structural breaks can affect the performance of forecasting methods in various ways. Therefore, understanding and comparing different models across these scenarios is essential.

**Objective:**

This study aimed to develop depression incidence forecasting models and compare the performance of autoregressive integrated moving average (ARIMA) and vector-ARIMA (VARIMA) and temporal fusion transformers (TFT) under different structural break scenarios.

**Methods:**

We developed population-wide depression incidence forecasting models and compared the performance of ARIMA and VARIMA-based methods to TFT-based methods. Using monthly depression incidence from 2002 to 2022 in Hong Kong, we applied sliding windows to segment the whole time series into 72 ten-year subsamples. The forecasting models were trained, validated, and tested on each subsample. Within each 10-year subset, the first 7 years were used for training, with the eighth year for setting hold-out validation, and the ninth and tenth years for testing. The accuracy of the testing set within each 10-year subsample was measured by symmetric mean absolute percentage error (SMAPE).

**Results:**

We found that in subsamples without significant slope or trend change (structural break), multivariate TFT significantly outperformed univariate TFT, vector-ARIMA (VARIMA), and ARIMA, with an average SMAPE of 11.6% compared to 13.2% (*P*=.01) for univariate TFT, 16.4% (*P*=.002) for VARIMA, and 14.8% (*P*=.003) for ARIMA. Adjusting for the unemployment rate improved TFT performance more effectively than VARIMA. When fluctuating outbreaks happened, TFT was more robust to sharp interruptions, whereas VARIMA and ARIMA performed better when incidence surged and remained high.

**Conclusions:**

This study provides a comparative evaluation of TFT and ARIMA and VARIMA models for forecasting depression incidence under various structural break scenarios, offering insights into predicting disease burden during both stable and unstable periods. The findings support a decision-making framework for model selection based on the nature of disruptions and data characteristics. For public health policymaking, the results suggest that TFT may be a more suitable tool for disease burden forecasting during periods of stable burden level or when sudden temporary interruption, such as pandemics or socioeconomic variation, impacts disease occurrence.

## Introduction

Depression is a major disease burden with substantial unmet care needs, necessitating urgent attention in health care planning. The Global Burden of Disease study indicates that the incidence of depression continuously increased from 2010 to 2019, reaching 274.8 million patients with an incidence of 3551.6 per 100,000 population in 2019 [1]. This burden has further increased since the COVID-19 pandemic rising by 20% to 30% [2,3]. The rising incidence and prolonged disease duration impose a significant strain on the health care system and create an economic burden on society, highlighting the importance of health policy planning for depression management [4]. A systematic review showed that the use of health care resources and the productivity loss or reduction of adult patients with depression are 2.6 times and 2.3 times that of nondepressed patients, respectively [5]. In addition, the World Health Organization reported insufficient spending on mental health over the total government health expenditure and called for estimating, planning, and monitoring the expenditure on mental health services over the long term [6]. An accurate projection of disease burden is essential for the planning of health care resources and improving the preparedness of health care systems to respond to foreseeable demand [7].

Population-wide depression incidence is sensitive to abrupt social stressors. For example, events such as pandemics [2,8-10], economic crises [11-13], social movement [14], and natural disasters [15] have been associated with observed surges in the incidence and prevalence of depression. These isolated historical events represent inherently unpredictable factors that create complex interruption scenarios, influencing the performance of incidence forecasting methods. Depression incidence is also affected by various demographic factors (eg, the elderly group is associated with a higher risk of depression) [16], environmental factors (eg, seasonality and temperature) [10], and socioeconomic variation [17]. Among this, unemployment has been reported as the most significant external risk factor for depression [12,18] and also presents a bidirectional positive association with depression [19,20]. Research gaps exist in the current modeling literature on population-based depression incidence forecasting, particularly in evaluating the impact of various interruption scenarios caused by historical events on model performance, and in effectively incorporating predictable external factors such as unemployment and seasonality.

The temporal fusion transformer (TFT) is a deep learning–based time-series forecasting tool recently seen in clinical applications used for health outcomes prediction, such as predicting population-level emergency attendance [21], individual-level vital sign trajectories [22], blood glucose [23], and hypertension [24]. TFT has been shown to outperform a set of competing methods in multihorizon time-series forecasting and the handling of structural breaks in time series [25]. The well-known and widely used vector autoregressive integrated moving average (VARIMA) models, the multivariate version of autoregressive integrated moving average (ARIMA), enables adjustment for historical data, random effects, and external covariates. VARIMA also enables the output variables to adjust to each other using a simultaneous equations approach to forecasting cointegrated systems of correlated variables [26]. It has been proven that VARIMA is effective in forecasting hospital and emergency admissions [27,28].

In this study, we used a population-based electronic medical records (EMRs) database in Hong Kong and developed TFT and VARIMA models for depression incidence forecasting, taking incidence and unemployment rates as dual outcomes for mutual adjustment. We aimed to evaluate the performance of these models in the presence of unpredictable isolated events such as pandemics, economic crises, and social movements and identify fit-for-purpose models for medium disease burden forecasting.

## Methods

**Figure 1:**
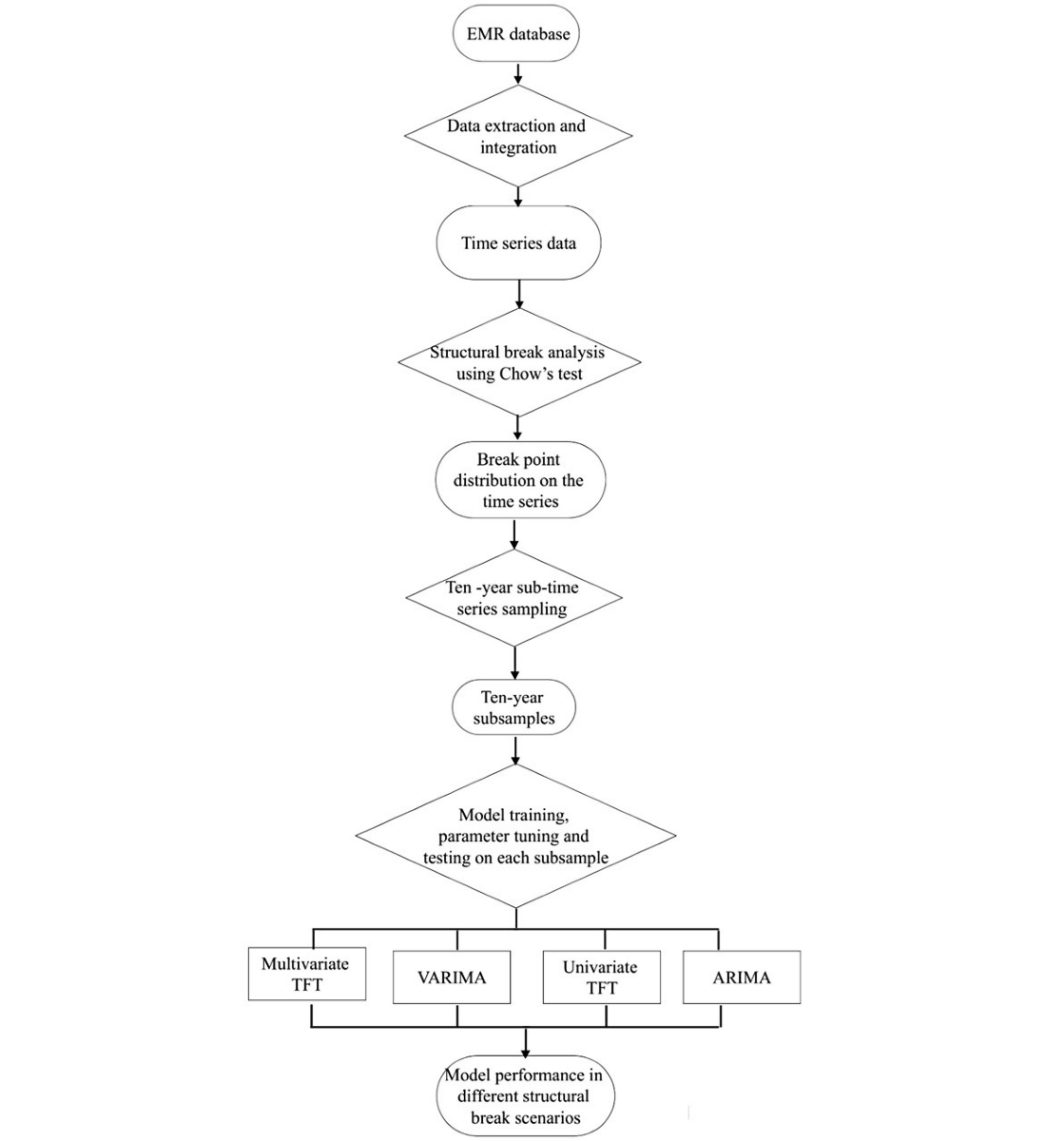
Diagram of the streamlined analytical plan for the comparative study of forecasting models. ARIMA: Auto-Regressive Integrated Moving Average; EMR: electronic medical records; Multivariate TFT: Multivariate Temporal Fusion Transformers; SMAPE: symmetric mean absolute percentage error; Univariate TFT: Univariate Temporal Fusion Transformers; VARIMA: Vector Auto-Regressive Integrated Moving Average.

The analytical framework for the comparative components of different forecasting models is illustrated in Figure 1.

### Data Source and Extraction

**Figure 2:**
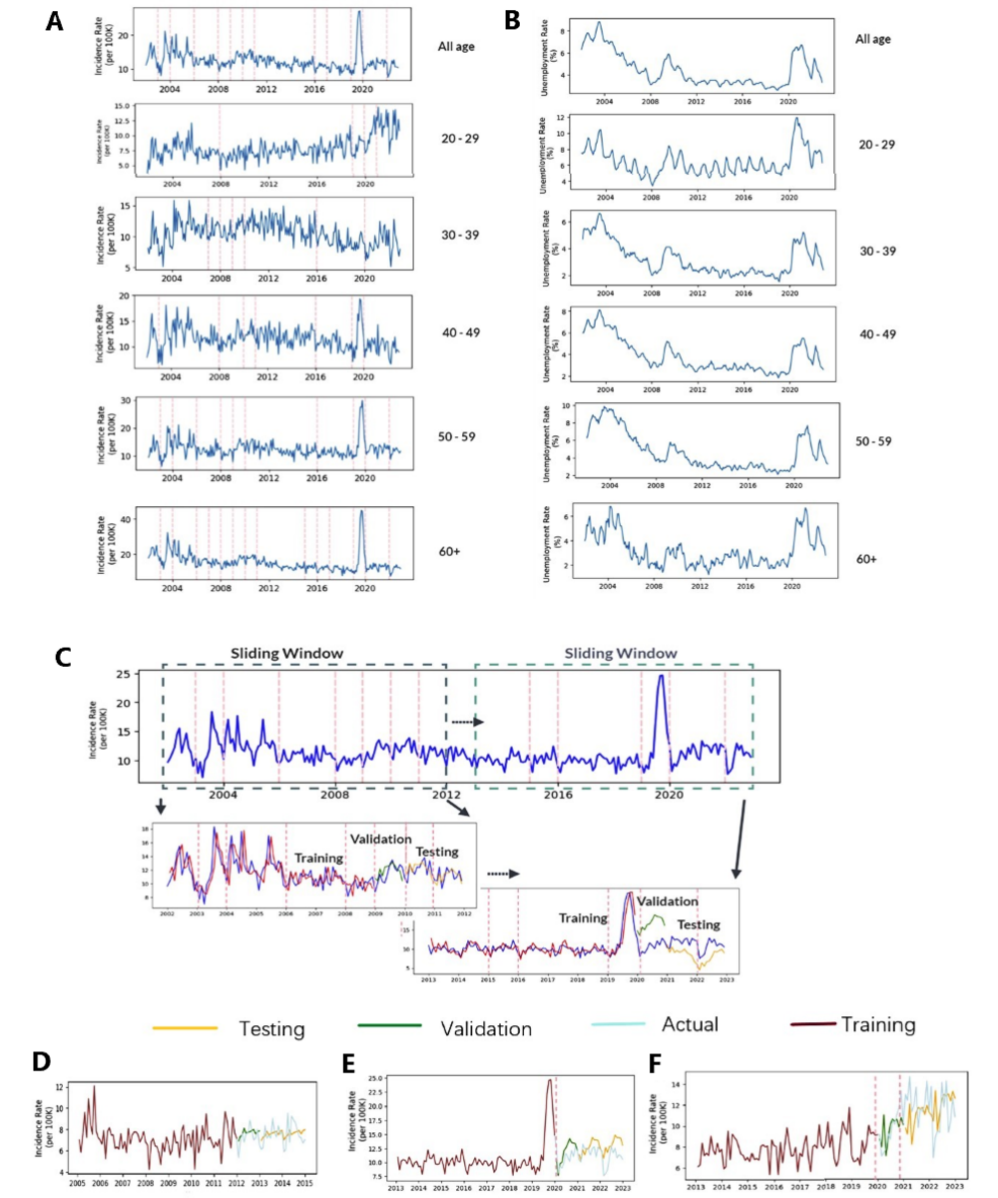
Depression incidence and unemployment rate in Hong Kong between 2002 and 2023. (A) Depression incidence time series for the overall population (age-standardized) and for specific age subgroups: vertical pink dotted lines represent the breakpoints on the timeseries as indicated by Chow’s test. (B) Unemployment rate time series for the overall 20 years population and for specific age subgroups. (C) Ten-year sub-timeseries sample set construction: segmenting the 10-year sub-timeseries (one window) according to year-by-year sliding. In the example shown for 2002-2022, there are 12 sliding windows in total. The first 7 years in each sub-timeseries is the training set, the eighth year is the validation set, and the ninth and tenth year are the testing set. In the database used in this study, we analyzed 72 sub-timeseries datasets (12 samples×6 groups) from the overall population and age subgroups. (D) An example of stable period sample. (E) An example of unstable period sample with sharp interruptions. (F) An example of unstable period sample with level shift.

The depression diagnosis data source was the Clinical Data Analysis and Reporting System (CDARS) managed by the Hospital Authority of Hong Kong. CDARS is a territory-wide EMR database covering more than 7 million eligible residents, which encompasses all publicly funded health care services and manages 76% of chronic medical conditions, including mental health issues [29]. The database has been used in multiple epidemiological depression studies and demonstrated its data authenticity and integrity [3,7,30-32]. New diagnosis records were identified by diagnosis codes (*ICD-9-CM* [*International Classification of Diseases, 9th Revision, Clinical Modification*]: 296.2, 300.4, 311) and ascertained by backtracking the database to 1993 to ensure that incident cases were free from depression before the first diagnosis. We extracted records to confine the analysis to the population aged 20 years with a new clinical diagnosis of depression between 2002 and 2022. Clinically diagnosed depression incidence in the overall population (age-standardized) and age subgroups (20-29, 30-39, 40-49, 50-59, 60+ years) were computed, using 2022 as the reference year (Figure 2A). Incidence is defined as the proportion of new cases occurring within a specific age group over a given period, with the denominator being the total population of that age group. Unemployment rates in each age subgroup and the overall population (Figure 2B) were obtained from the Census and Statistics Department of Hong Kong Government [33]. Monthly data granularity was selected to balance the need for sufficient data points to train and validate the models while effectively capturing meaningful trends, including seasonality [3]. This choice also helps ease sparsity issues that can arise in overly granular data, such as daily data, where zero records would occur.

### Time-Series Analysis to Identify Structural Breaks

We applied Chow test breakpoint analysis [34] to detect structural breaks in the time series, which indicates whether the slopes and intercepts of outcomes in two adjacent time periods are identical [34]. We conducted the test for each pair of adjacent years (each year comprised 12 data points from January to December) to test for structural breaks. When the null hypothesis of the Chow test was accepted, we assumed that the adjacent 2 years are coherent.

### Construction of TFT and VARIMA Models

We developed multivariate forecasting models using VARIMA and multivariate TFT, both adjusting for seasonality as a covariate. Both unemployment rates and depression incidence were incorporated as simultaneous outcomes. As an indicator of socioeconomic stress, the unemployment rates are commonly or generally known to be nonstationary and related to socioeconomic variation, which makes them not directly predictable. Social epidemiological theories can account for bidirectional causation between population unemployment rates and depression incidence. Both TFT [25] and VARIMA models can incorporate bidirectional adjustment between the synchronous and lagged values of these 2 outcomes. We used the seasonal component of additive decomposition for seasonality adjustment [35], and a set of dummy variables to label months as independent covariates. Univariate TFT and ARIMA models that took depression incidence as the single output variable were also fitted for model comparison. The testing accuracies of the various models on all the 10-year sub-timeseries samples were measured by symmetric mean absolute percentage error (SMAPE) between the forecasted results and actual value. Features of all the models are shown in Table S1 in Multimedia Appendix 1.

### Model Training, Parameter Tuning, and Output Validation

“VARMAX” and “ARIMA” functions from the “statsmodels.tsa” package in Python were used for VARIMA and ARIMA model training. An autocorrelation function plot was generated for all the training sets of the 10-year sub-time series to test for data stationarity. Combining the Kwiatkowski-Phillips-Schmidt-Shin tests and augmented Dickey-Fuller test, we determined that both depression and unemployment were integrated of order 1 and first-order differencing applied to both variables. We then tested for significant autocorrelation in the first to third data lags. To avoid overfitting, the combination of the orders of the autoregressive and moving average components were set in the range from 0 to 3 and from 0 to 2, respectively. The trend parameter had two options, “n” and “c,” where “n” represents no drift and “c” represents constant drift in the model.

The TFT models were constructed using the “TemporalFusionTransformer” package developed by Pytorch (an open-source machine learning framework trademarked by the Linux Foundation). To optimize model performance, we used another package called “Optuna” to fine-tune various settings such as how much data the model processes at once, the speed of learning, the size of different parts of the model, how often to drop parts of the model to prevent overfitting, and other important parameters (Table S2 in Multimedia Appendix 1). The validation and testing accuracy were measured by SMAPE for both ARIMA and VARIMA and TFT models, thus enabling a fair comparison between the 2 methods. To ensure consistency between the training and validation steps, the TFT models were trained using the SMAPE loss function. This loss function proved beneficial for point forecasting, symmetric error evaluation, and providing easily interpretable and comparable performance metrics. To prevent overfitting and improve model generalization, we used dropout regularization and gradient clipping during training, along with the Adam optimizer and a learning rate schedule that reduces the learning rate when improvements stop. Furthermore, we implemented an early stopping mechanism that monitors the validation loss and stops training if the loss does not improve for a specified number of consecutive epochs.

### Ethical Considerations

This study received ethics approval from the institutional review board of The University of Hong Kong Hospital Authority Hong Kong Western Cluster (UW 20-218). Patient identification was anonymized to protect confidentiality and patient consent was not required. No participant compensation was provided for the study.

## Results

### Overview

During the study period from 2002 to 2022, we identified 173,865 patients newly diagnosed with depression aged 20 years or older. The mean age of these patients was 51.6 (SD 17.7) years and 72% (125,183/173,865) were female. As shown in Figure 2A, the incidence of depression was aggregated for the overall population (age-standardized) as well as for specific age subgroups (20-29, 30-39, 40-49, 50-59, 60+ years). The corresponding unemployment rates were also collected, as depicted in Figure 2B.

### Chow Test to Identify Structural Breaks

According to structural break analysis based on Chow test, the incidence patterns between 2002 and 2022 differed between age groups. For example, the subgroup aged 20-29 years had stable depression incidence before 2018 with only one breakpoint identified in January 2008 (Figure 2A). However, breakpoints occurred more frequently in other age groups during the same period. From 2019 to 2022, depression incidence across the whole population and all age subgroups experienced significant fluctuations, and January 2020 (corresponding to the start of the COVID-19 pandemic) became the breakpoint for all the time series. In the subgroups aged 40-49, 50-59, and 60 years, sharp interruptions occurred in 2019 (corresponding to the major social movement period in Hong Kong), leading to outliers in the time series.

By applying a 10-year sliding window segmentation to generate overlapping subsamples of 10 consecutive years (2002-2011, 2003-2012, etc) for each of the 6 age subgroups (Figure 2C), we identified 72 ten-year subsamples. In each subsample, the models were trained on data for the first 7 years (training set). The corresponding parameters of the forecasting models were refined based on the model’s accuracy in predicting outcomes in the eighth year (independent validation set), and the performance of the models was evaluated using outcomes in the ninth and tenth years (testing set). In each 10-year subsample, Chow test breakpoint analysis was performed between the seventh and eighth year, eighth and ninth year, and ninth and tenth year. The distribution of breakpoints is characterized by the coherence of the training, validation, and testing set in the subsamples. When there was no breakpoint in between, we assumed that the validation and testing sets were a smooth extension of the training set. Models were assumed to be trained to forecast incidence in a “stable period.” An example of a stable period sample is shown in Figure 2D, in which no breakpoint existed throughout the last 4 years (from 2011 to 2014). On the other hand, if one or more breakpoints existed in the last 4 years, such a sliding window was treated as an “unstable period” sample. Figure 2E is an example of an unstable period sample with a sharp interruption where January 2020 was a breakpoint. Figure 2F is an example of an unstable period sample with a level shift in which January 2020 and January 2021 were identified as breakpoints. Among all the 10-year subsamples, 53 were subsamples classified as unstable periods within our dataset (Table S3 in Multimedia Appendix 1).

### Performance Comparison Between TFT and VARIMA and ARIMA Models

**Figure 3:**
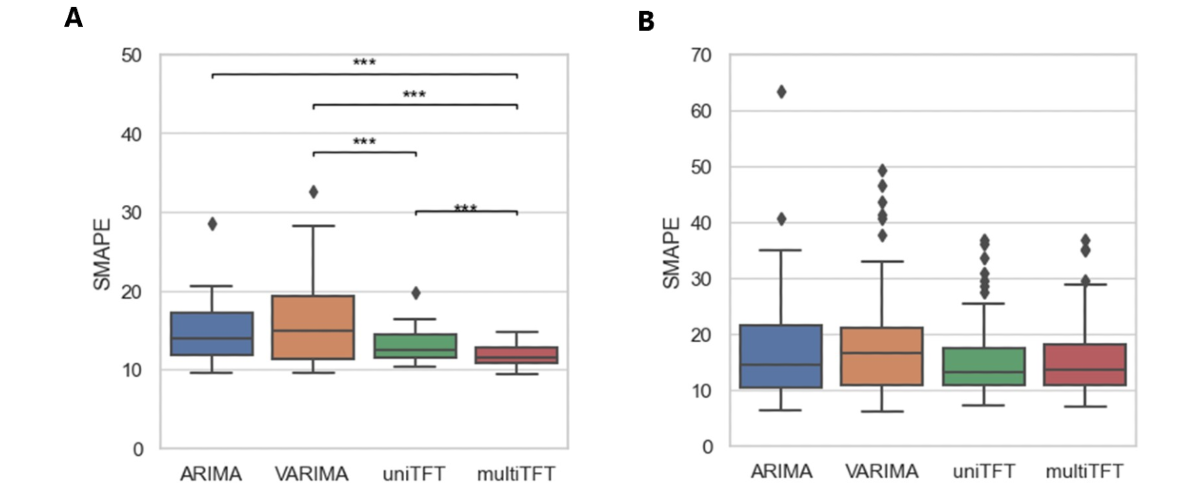
Testing accuracy comparison between models. (A) Stable periods with no breakpoint between training, validation, and testing periods. (B) Unstable periods with one or more breakpoints between training, validation, and testing periods. ARIMA: autoregressive integrated moving average; multiTFT: multivariate temporal fusion transformers; SMAPE: symmetric mean absolute percentage error; uniTFT: univariate temporal fusion transformers; VARIMA: vector autoregressive integrated moving average.

In the set of stable period samples, multivariate TFT models significantly outperformed the univariate TFT, VARIMA, and ARIMA. Among stable periods, *t* tests comparing the SMAPE of these 4 models (Figure 3) showed that the average SMAPE at 11.6% for multivariate TFT was significantly lower than the SMAPE for univariate TFT at 13.2% (*P*=.01), for VARIMA at 16.4% (*P*=.002), and for ARIMA at 14.8% (*P*=.003) of the same data. Univariate TFT also outperformed VARIMA (*P*=.03), while there was no significant difference between VARIMA and ARIMA. For the set of unstable period samples, these 4 methods had no significant difference on the overall testing accuracy. The average SMAPE of multivariate TFT was at 15.9%, univariate TFT was at 15.8%, VARIMA was at 18.3%, and ARIMA was at 17.5%.

### Performance Comparison During Periods With Breakpoints

**Figure 4:**
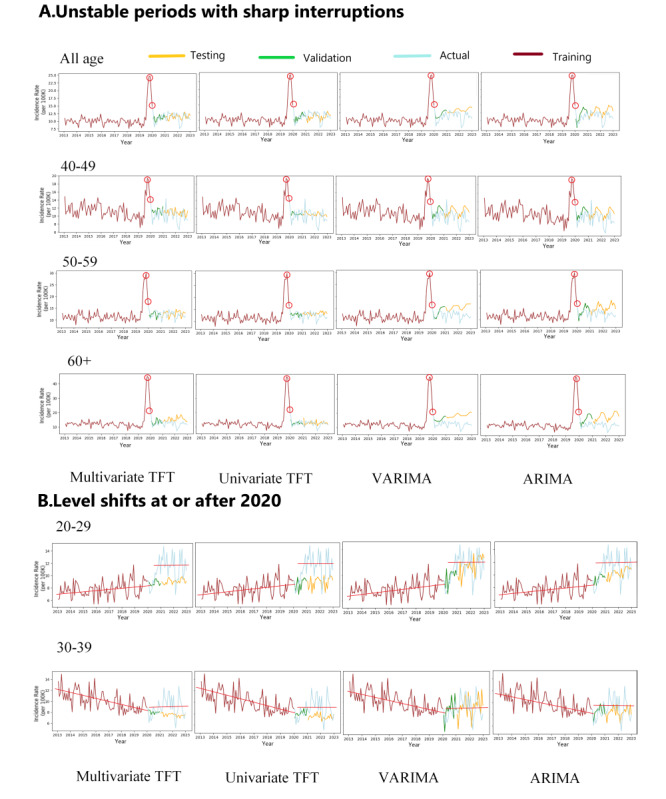
Model performance comparison during unstable periods with a sharp interruption or level shift. Training set: 2013-2019; validation set: 2020; testing set: 2021-2022. (A) Model performance comparison during unstable periods with a sharp interruption in 2019: the red circles highlight the last points in the training set, which heavily influenced the prediction output of the ARIMA/VARIMA models due to their autoregression mechanism. These points fall within the sharp interruption period of 2019. (B) Model performance comparison during unstable period with level shift at or after 2020: the red lines indicate the changes in levels occurring at or after 2020. ARIMA: autoregressive integrated moving average; multivariate TFT: multivariate temporal fusion transformers; univariate TFT: univariate temporal fusion transformers; VARIMA: vector autoregressive integrated moving average.

Due to the high variation of accuracy in the set of unstable period subsamples, specific manifestations of structural break should be considered. Taking the sub–time series in the period of 2013-2022 as an example, significant performance differences occurred among the VARIMA and ARIMA and TFT models. As shown in Figure 4, from 2019 to 2022, structural breakpoints in the time series of depression incidence occurred, representing a consistent 4-year ongoing population shock. We observed the response of different age groups to the interruption in different patterns. Incidence in age groups 40-49, 50-59, and 60+ years showed a sharp increase in 2019 (Figure 4A). After 2019, the incidence stabilized. However, for younger age groups (20-29 and 30-39 years), no sharp increase occurred in 2019, but a significant level shift was found in the 20-29 and 30-39 age subgroups in 2020 (Figure 4B).

Table 1 revealed that the performance of TFT and VARIMA and ARIMA models during the unstable period showed sharp increases. Comparison forecasts using multivariate and univariate TFT to VARIMA and ARIMA models indicated that the latter forecast higher depression incidence than observed, resulting in inaccurate estimates with large SMAPE (15.3%-43.6% for VARIMA and ARIMA models vs 11.1%-25.7% of TFT models). The performance of different models during the unstable period with level shifts was also compared (Table 1). The VARIMA and ARIMA models significantly outperformed the multivariate and univariate TFT models by capturing the level shift patterns by training and validation process with SMAPE of 16.2%-18.8% versus 23.1%-27.6%, respectively.

**Table 1:** Model performance comparison during different structural break scenarios between 2013 and 2022 (on the 10-year sub–time series samples between 2013 and 2022).

Age group (years)	Structural break scenario	SMAPE^a^			
		VARIMA^b^	ARIMA^c^	Multivariate TFT^d^	Univariate TFT^e^
All	Sharp interruption	16.8	15.3	12.1	11.1^f^
40-49	Sharp interruption	17.9	17	16.1	15^f^
50-59	Sharp interruption	25.8	21.6	13.6	13^f^
60+	Sharp interruption	43.6	40.7	25.7	13.3^f^
20-29	Level shift	17.8^f^	18.6	27.6	25.8
30-39	Level shift	18.4	16.2^f^	23.1	23.2

^a^SMAPE: symmetric mean absolute percentage error**.**

^b^VARIMA: vector autoregressive integrated moving average.

^c^ARIMA: autoregressive integrated moving average.

^d^Multivariate TFT: multivariate temporal fusion transformers.

^e^Univariate TFT: univariate temporal fusion transformers.

^f^Lowest SMAPEs.

## Discussion

This study sought to answer the research question “How do deep learning–based TFT models compare to regression-based ARIMA and VARIMA models for forecasting the medium-term incidence of depression, particularly under varying structural break scenarios such as stable periods and periods with abrupt disruptions?” We discovered that in the stable periods in which there was no breakpoint between training, validation, and testing datasets, multivariate TFT significantly outperformed the univariate TFT, VARIMA, and ARIMA in incidence forecasting. This finding not only indicated that in stable periods, TFT was a better choice to forecast depression incidence than ARIMA, but also suggested the effectiveness and necessity of adjusting for unemployment rates in both models to improve accuracy. The multivariate TFT model presented the ability to partially capture the bidirectional relationship between depression incidence and unemployment, showcasing its potential to model complex interactions between socioeconomic factors and health outcomes. However, in unstable periods, the multivariate TFT did not significantly outperform the univariate TFT. On the other hand, VARIMA did not statistically outperform ARIMA both in stable and unstable periods, which indicated that the vector autoregression-based linear model might not significantly improve incidence forecasting by adjusting for the unemployment rate.

In subsamples that had one or more breakpoints between the training, validation, and testing set, the presence and number of breakpoints varied between different time periods and age groups. Therefore, a general overall comparison could not distinguish performance differences between TFT and VARIMA and ARIMA in different structural break scenarios. After further exploration of the models’ performance in different interrupted time periods, we found that TFT models were more robust to outliers, such as abrupt shocks in the time series, where incidence surged and then returned to its previous level in a short period of time. On the other hand, ARIMA and VARIMA were more sensitive to level shifts, in which the depression incidence remained high after an increase.

These phenomena could be due to the modeling mechanisms of the TFT and ARIMA models. VARIMA and ARIMA models are based on autoregression, which is a linear modeling approach that relies on past observations over specific time lags of the training set to make predictions. On the other hand, TFT models capture long-term dependencies and patterns in the data, which give rise to self-attention mechanisms modified from transformer-based architectures [25]. In our examples shown in Figure 4A, incidence in 2019 was an outlier in the training set. Because of autoregression and first-order differencing in VARIMA and ARIMA models, the 2019 data points contributed to the time-lagged terms in the model and hence to the forecasting result. Since these time points were still in the structural break (outlier) period, this often led to an overestimation of incidence. However, the multivariate and univariate TFT models were more robust to the outliers and the forecasted results were closer to the observed incidence. In the examples of level shift shown in Figure 4B, because the VARIMA and ARIMA models were more sensitive to recent time points and the trend parameter was adjusted based on the accuracy of the validation set, the models were better able to detect sudden changes in the time series. Therefore, the VARIMA and ARIMA models could outperform the TFT models in scenarios with level shifts.

This modeling study is the first attempt at comparing the performance of TFT and ARIMA methods in forecasting disease incidence, and we introduce a practical framework for model evaluation and selection, using a sliding window approach to systematically assess model performance across various structural break scenarios. As shown in Figure 1, such a study design can guide the selection of models for forecasting disease burden in stable periods and times of major events with uncertain effects on disease epidemiology, especially in complex scenarios involving population shocks, such as the pandemic and sociopolitical events. The repeated occurrence of stable and disrupted periods along the time axis in various age subgroups strengthens the applicability of our comparative study’s findings to diverse structural break scenarios. Putting this into practice for model selection, TFT models would be more appropriate for stable periods or events that end relatively quickly. For events with long-term effects, such as level shifts, ARIMA models would be a better option.

The study also has limitations. While contributing to state-of-the-art model selection, we primarily focused solely on one disease, choosing a disease with a complex time series containing breakpoints and abrupt changes. However, further research is warranted to explore the applicability and performance of these models in other types of time series or disease areas, such as communicable diseases and long-term projections. To account for external risk factors, the unemployment rate was selected as the second outcome in the multivariate models due to its bidirectional effect on depression and its timely monthly availability. Nevertheless, future studies should incorporate additional factors such as socioeconomic and health care accessibility data to further refine the models. In addition, our dataset was limited to health care records from Hong Kong, which may not fully generalize to other populations. Differences in health care–seeking behavior and socioeconomic characteristics between public and private health care users may influence depression incidence patterns. Future research could focus on validating and adapting our models in other populations and applying the model evaluation framework to diverse datasets and different patient subgroups, to assess the generalizability of our findings and refine the models for broader applicability. Furthermore, hybrid modeling approaches that integrate ARIMA and TFT could be explored in future studies to leverage their complementary strengths, potentially enhancing forecasting performance across different scenarios. Such hybrid models may prove particularly valuable in addressing diverse time-series characteristics, including both stable and unstable periods.

## Appendix

Multimedia Appendix 1 Features of all the tested models; Parameter ranges for hyperparameter tuning of TFT model; Testing accuracy (SMAPE) and the breakpoint distribution between training, validation and testing set of each ten-year sub-timeseries; Feature importance (SHAP values) of independent covariates in the ARIMA/VARIMA models and feature importance (decoder variable importance) of independent covariates in the univariate TFT/multivariate TFT models of all age during unstable periods with a sharp interruption in 2019.

## Abbreviations

ARIMA: auto-regressive integrated moving average
CDARS: clinical data analysis and reporting system
EMR: electronic medical record
ICD-9-CM: International Classification of Diseases, 9th Revision, Clinical Modification
SMAPE: symmetric mean absolute percentage error
TFT: temporal fusion transformer
VARIMA: vector auto-regressive integrated moving average

## Data Availability

The data used in this study cannot be publicly shared due to third-party restrictions but are available upon request through an application to the Hong Kong Hospital Authority. The HK EHR database is managed by the Hong Kong Hospital Authority. Local academic institutions, government departments, or nongovernmental organizations can apply for access via the Hong Kong Hospital Authority Data Sharing Portal (detailed application procedures in [36]).
