# Supplementary material for: Population-Wide Depression Incidence Forecasting Comparing Autoregressive Integrated Moving Average and Vector Autoregressive Integrated Moving Average to Temporal Fusion Transformers: Longitudinal Observational Study
Source: J Med Internet Res. 2025 May 12;27:e67156. doi: 10.2196/67156 (PMC12107200; doi:10.2196/67156)
Supplement: Multimedia Appendix 1 [file jmir_v27i1e67156_app1.docx]

**Table S1.** Features of all the tested models.

|  | **Seasonality adjustment** | **Unemployment rate** | **Depression incidence** |
| --- | --- | --- | --- |
| Univariate TFT | Yes, as co-variate | No | Yes, as outcome |
| Multi-variate TFT | Yes, as co-variate | Yes, as outcome | Yes, as outcome |
| ARIMA | Yes, as co-variate | No | Yes, as outcome |
| VARIMA | Yes, as co-variate | Yes, as outcome | Yes, as outcome |

Note: ARIMA**:** Auto-Regressive Integrated Moving Average; VARIMA: Vector Auto-Regressive Integrated Moving Average**;** Multivariate TFT: Multivariate Temporal Fusion Transformers; Univariate TFT: Univariate Temporal Fusion Transformers.

**Table S2.** Parameter ranges for hyperparameter tuning of TFT model.

| **Name** | **Range** | **Explanation** |
| --- | --- | --- |
| batch_size | [32, 128] | No. of samples used in each training iteration |
| learning_rate | [2e-4, 1e-2] | Step size at each iteration while moving toward a minimum of a loss function |
| hidden_size | [16, 128] | No. of neurons of each layer of GRN (Should be a multiple of the number of attention head) |
| hidden_continuous_size | [16, hidden_size] | No. of neurons of each FC layer for continuous variables |
| dropout | [0.1, 0.3] | Fraction of neurons affected by dropout |
| attention_head_size | [1, 4] | No. of attention heads |

Note: TFT: Temporal Fusion Transformers; GRN: Gated Residual Networks; FC layer: Fully Connected layer.

**Table S3.** Testing accuracy (SMAPE) and the breakpoint distribution between training, validation and testing set of each ten-year sub-timeseries.

| **Period** | **Age subgroup** | **Break between training and validation** | **Break between validation and testing** | **Break between first testing year and second testing year** | **SMAPE of VARIMA** | **SMAPE of ARIMA** | **SMAPE of univariate TFT** | **SMAPE of multivariate TFT** | **Unstable period** |
| --- | --- | --- | --- | --- | --- | --- | --- | --- | --- |
| 2002.1 – 2011.12 | All | Y | Y | Y | 6.0 | 8.2 | 8.3 | 7.1 | Y |
| 2003.1 – 2012.12 | All | Y | Y | N | 12.9 | 8.4 | 10.5 | 8.3 | Y |
| 2004.1 – 2013.12 | All | Y | N | N | 6.9 | 6.6 | 9.9 | 9.4 | Y |
| 2005.1 – 2014.12 | All | N | N | N | 11.9 | 14.0 | 12.6 | 12.4 | N |
| 2006.1 – 2015.12 | All | N | N | Y | 9.4 | 6.7 | 10.8 | 8.6 | Y |
| 2007.1 – 2016.12 | All | N | Y | Y | 7.1 | 6.3 | 8.5 | 8.4 | Y |
| 2008.1 – 2017.12 | All | Y | Y | Y | 6.2 | 8.1 | 7.1 | 8.3 | Y |
| 2009.1 – 2018.12 | All | Y | N | N | 6.5 | 7.1 | 8.7 | 8.7 | Y |
| 2010.1 – 2019.12 | All | N | N | Y | 22.1 | 22.4 | 25.4 | 25.3 | Y |
| 2011.1 – 2020.12 | All | N | Y | Y | 25.6 | 24.4 | 28.7 | 28.7 | Y |
| 2012.1 – 2021.12 | All | Y | Y | N | 40.6 | 14.4 | 12.1 | 15.0 | Y |
| 2013.1 – 2022.12 | All | Y | N | Y | 16.8 | 15.3 | 11.1 | 12.1 | Y |
| 2002.1 – 2011.12 | 20-29 | N | N | N | 15.0 | 17.1 | 15.3 | 13.7 | N |
| 2003.1 – 2012.12 | 20-29 | N | N | N | 32.6 | 20.6 | 11.5 | 10.9 | N |
| 2004.1 – 2013.12 | 20-29 | N | N | N | 19.9 | 14.1 | 10.7 | 10.8 | N |
| 2005.1 – 2014.12 | 20-29 | N | N | N | 14.1 | 12.9 | 11.5 | 10.9 | N |
| 2006.1 – 2015.12 | 20-29 | N | N | N | 9.6 | 9.6 | 12.5 | 12.6 | N |
| 2007.1 – 2016.12 | 20-29 | N | N | N | 28.2 | 17.2 | 12.1 | 11.6 | N |
| 2008.1 – 2017.12 | 20-29 | N | N | N | 11.4 | 12.6 | 13.3 | 13.3 | N |
| 2009.1 – 2018.12 | 20-29 | N | N | N | 15.0 | 14.6 | 16.3 | 14.7 | N |
| 2010.1 – 2019.12 | 20-29 | N | N | Y | 16.0 | 16.2 | 15.5 | 16.2 | Y |
| 2011.1 – 2020.12 | 20-29 | N | Y | Y | 16.8 | 17.1 | 18.1 | 15.5 | Y |
| 2012.1 – 2021.12 | 20-29 | Y | Y | Y | 18.6 | 16.2 | 33.6 | 36.8 | Y |
| 2013.1 – 2022.12 | 20-29 | Y | Y | N | 17.8 | 18.6 | 27.6 | 25.8 | Y |
| 2002.1 – 2011.12 | 30-39 | Y | Y | N | 7.5 | 8.2 | 9.3 | 9.9 | Y |
| 2003.1 – 2012.12 | 30-39 | Y | N | N | 9.1 | 8.9 | 10.2 | 10.1 | Y |
| 2004.1 – 2013.12 | 30-39 | N | N | N | 10.8 | 10.6 | 11.2 | 12.0 | N |
| 2005.1 – 2014.12 | 30-39 | N | N | N | 11.1 | 11.0 | 15.2 | 11.2 | N |
| 2006.1 – 2015.12 | 30-39 | N | N | N | 12.2 | 12.4 | 12.3 | 12.8 | N |
| 2007.1 – 2016.12 | 30-39 | N | N | Y | 10.3 | 10.4 | 17.3 | 16.5 | Y |
| 2008.1 – 2017.12 | 30-39 | N | Y | N | 22.0 | 20.9 | 13.2 | 12.6 | Y |
| 2009.1 – 2018.12 | 30-39 | Y | N | N | 18.4 | 18.5 | 13.3 | 15.0 | Y |
| 2010.1 – 2019.12 | 30-39 | N | N | N | 15.1 | 13.7 | 13.6 | 9.8 | N |
| 2011.1 – 2020.12 | 30-39 | N | N | Y | 13.6 | 14.5 | 12.6 | 18.2 | Y |
| 2012.1 – 2021.12 | 30-39 | N | Y | N | 27.7 | 13.7 | 15.6 | 14.4 | Y |
| 2013.1 – 2022.12 | 30-39 | Y | N | N | 18.4 | 16.2 | 23.1 | 23.2 | Y |
| 2002.1 – 2011.12 | 40-49 | N | Y | Y | 10.1 | 11.4 | 11.8 | 9.5 | Y |
| 2003.1 – 2012.12 | 40-49 | Y | Y | N | 11.4 | 10.8 | 14.5 | 13.2 | Y |
| 2004.1 – 2013.12 | 40-49 | Y | N | N | 13.3 | 11.3 | 15.2 | 13.9 | Y |
| 2005.1 – 2014.12 | 40-49 | N | N | N | 11.1 | 11.2 | 11.5 | 10.0 | N |
| 2006.1 – 2015.12 | 40-49 | N | N | N | 18.8 | 18.3 | 14.7 | 11.3 | N |
| 2007.1 – 2016.12 | 40-49 | N | N | Y | 10.7 | 10.6 | 12.4 | 11.0 | Y |
| 2008.1 – 2017.12 | 40-49 | N | Y | N | 13.5 | 13.6 | 15.6 | 17.7 | Y |
| 2009.1 – 2018.12 | 40-49 | Y | N | N | 16.5 | 15.9 | 12.5 | 12.5 | Y |
| 2010.1 – 2019.12 | 40-49 | N | N | Y | 21.0 | 20.9 | 22.5 | 21.4 | Y |
| 2011.1 – 2020.12 | 40-49 | N | Y | Y | 19.9 | 26.4 | 20.7 | 20.5 | Y |
| 2012.1 – 2021.12 | 40-49 | Y | Y | N | 13.3 | 19.9 | 20.5 | 11.1 | Y |
| 2013.1 – 2022.12 | 40-49 | Y | N | N | 17.9 | 17.0 | 15.0 | 16.1 | Y |
| 2002.1 – 2011.12 | 50-59 | Y | Y | N | 20.2 | 22.8 | 11.1 | 12.3 | Y |
| 2003.1 – 2012.12 | 50-59 | Y | N | N | 13.9 | 13.0 | 12.1 | 12.8 | Y |
| 2004.1 – 2013.12 | 50-59 | N | N | N | 10.1 | 9.7 | 10.4 | 10.3 | N |
| 2005.1 – 2014.12 | 50-59 | N | N | N | 17.3 | 17.3 | 14.0 | 12.9 | N |
| 2006.1 – 2015.12 | 50-59 | N | N | N | 25.7 | 28.6 | 11.3 | 11.5 | N |
| 2007.1 – 2016.12 | 50-59 | N | N | Y | 12.2 | 10.2 | 14.3 | 11.7 | Y |
| 2008.1 – 2017.12 | 50-59 | N | Y | N | 10.3 | 10.0 | 9.9 | 9.5 | Y |
| 2009.1 – 2018.12 | 50-59 | Y | N | N | 11.0 | 8.4 | 10.8 | 11.1 | Y |
| 2010.1 – 2019.12 | 50-59 | N | N | Y | 25.4 | 24.2 | 29.4 | 26.7 | Y |
| 2011.1 – 2020.12 | 50-59 | N | Y | Y | 37.7 | 30.2 | 30.9 | 29.5 | Y |
| 2012.1 – 2021.12 | 50-59 | Y | Y | N | 17.9 | 14.1 | 10.5 | 9.9 | Y |
| 2013.1 – 2022.12 | 50-59 | Y | N | Y | 25.8 | 21.6 | 13.0 | 13.6 | Y |
| 2002.1 – 2011.12 | 60+ | Y | Y | Y | 12.2 | 11.0 | 13.4 | 8.5 | Y |
| 2003.1 – 2012.12 | 60+ | Y | Y | N | 46.6 | 30.9 | 17.4 | 19.8 | Y |
| 2004.1 – 2013.12 | 60+ | Y | N | N | 49.2 | 63.4 | 15.0 | 11.3 | Y |
| 2005.1 – 2014.12 | 60+ | N | N | N | 21.0 | 16.9 | 19.8 | 9.4 | N |
| 2006.1 – 2015.12 | 60+ | N | N | Y | 18.7 | 9.4 | 9.5 | 15.9 | Y |
| 2007.1 – 2016.12 | 60+ | N | Y | Y | 18.3 | 33.9 | 10.1 | 15.5 | Y |
| 2008.1 – 2017.12 | 60+ | Y | Y | Y | 11.7 | 13.6 | 12.4 | 12.8 | Y |
| 2009.1 – 2018.12 | 60+ | Y | Y | N | 10.9 | 11.3 | 11.8 | 15.4 | Y |
| 2010.1 – 2019.12 | 60+ | Y | N | Y | 32.8 | 33.3 | 36.1 | 34.9 | Y |
| 2011.1 – 2020.12 | 60+ | N | Y | Y | 41.4 | 35.0 | 36.9 | 35.3 | Y |
| 2012.1 – 2021.12 | 60+ | Y | Y | N | 10.0 | 26.8 | 10.1 | 10.8 | Y |
| 2013.1 – 2022.12 | 60+ | Y | N | Y | 43.6 | 40.7 | 13.2 | 25.7 | Y |

Note: SMAPE: symmetric mean absolute percentage error**;** ARIMA**:** Auto-Regressive Integrated Moving Average; VARIMA: Vector Auto-Regressive Integrated Moving Average**;** Multivariate TFT: Multivariate Temporal Fusion Transformers; Univariate TFT: Univariate Temporal Fusion Transformers

**Figure S1.** Feature importance (SHAP values) of independent covariates in the ARIMA/VARIMA models and feature importance (decoder variable importance) of independent covariates in the univariate TFT/multivariate TFT models of all age during unstable periods with a sharp interruption in 2019.


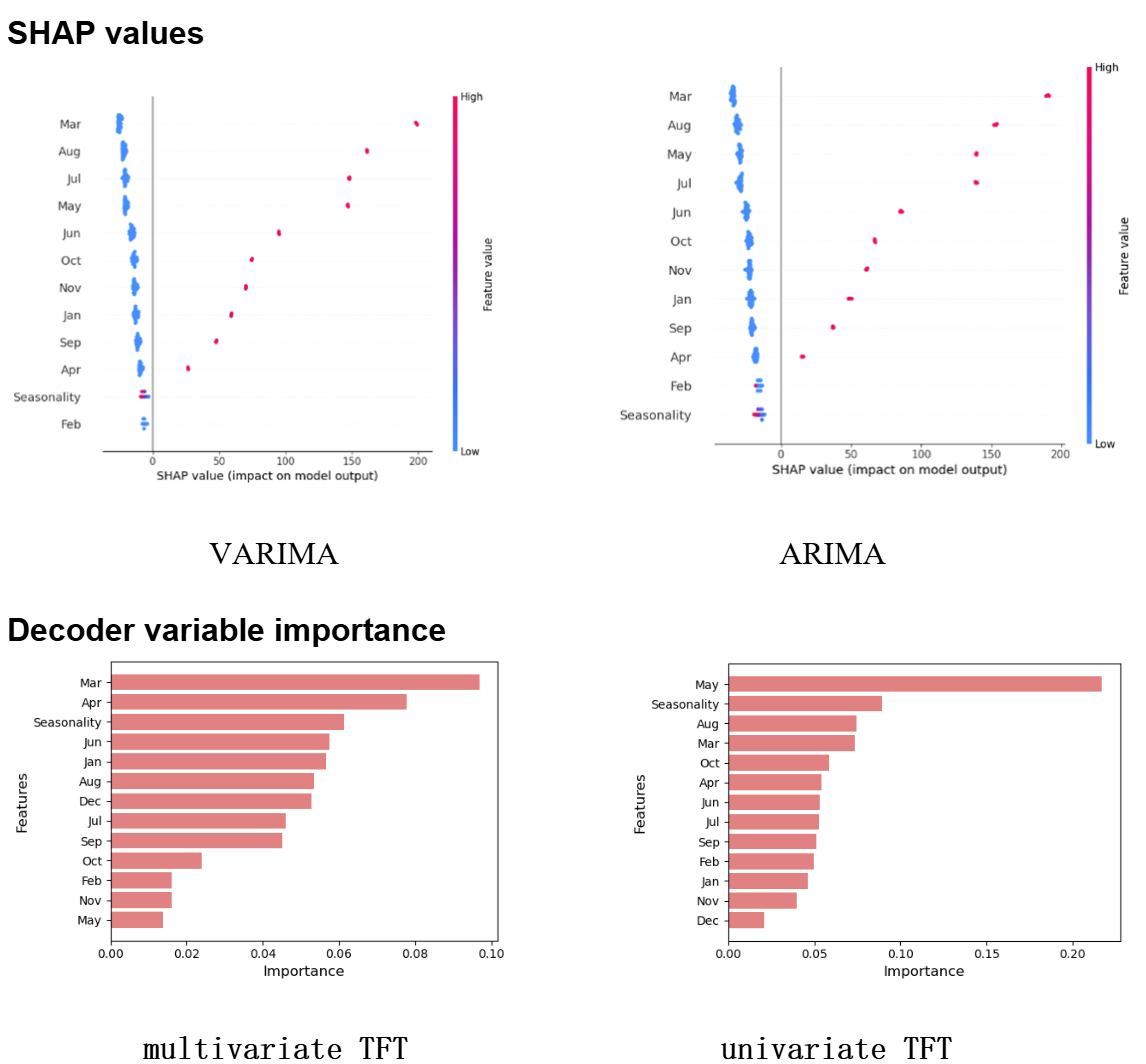


Note: SHAP value: SHapley Additive exPlanations value; ARIMA: Auto-Regressive Integrated Moving Average; VARIMA: Vector Auto-Regressive Integrated Moving Average; Multivariate TFT: Multivariate Temporal Fusion Transformers; Univariate TFT: Univariate Temporal Fusion Transformers
